# Supplementary material for: A Self-Assembling Pfs230D1-Ferritin Nanoparticle Vaccine Has Potent and Durable Malaria Transmission-Reducing Activity
Source: Vaccines (Basel). 2024 May 16;12(5):546. doi: 10.3390/vaccines12050546 (PMC11125772; doi:10.3390/vaccines12050546)
Supplement: Supplementary file 1 [file vaccines-12-00546-s001.zip › vaccines-2986310-supplementary.pdf]

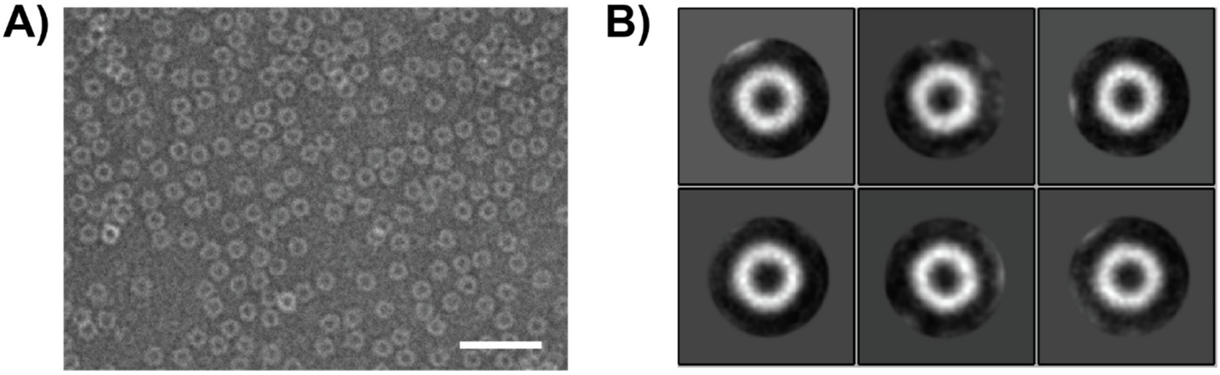

Figure S1. Ferritin forms a nanoparticle. (A) Negative-stain electron microscopy image of ferritin, scale bar = 50 nm, with (B) 2D classification averages.

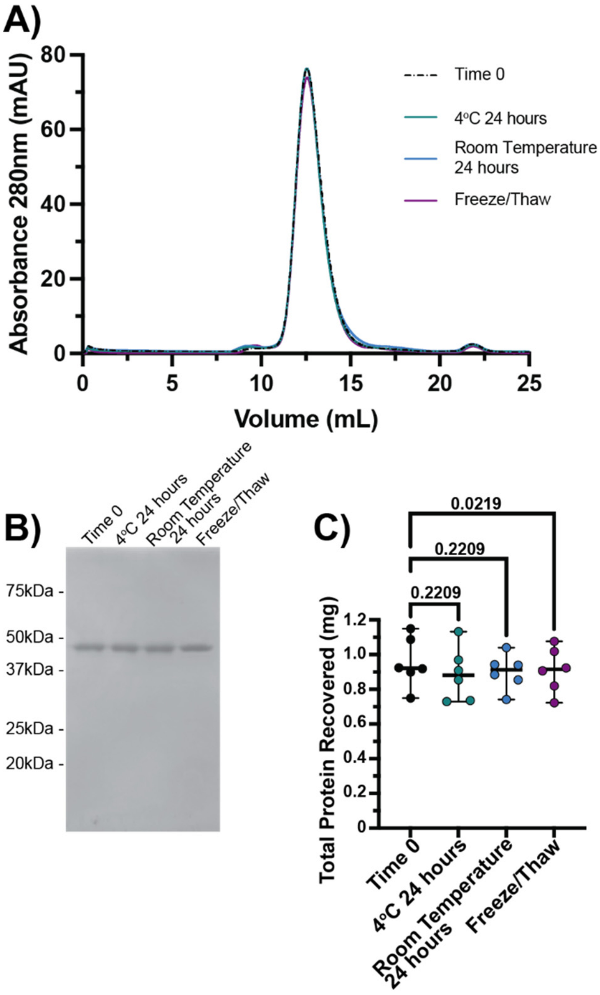

Figure S2. Pfs230D1-ferritin is stable under multiple conditions. (A) One representative experiment of the size exclusion purification of Pfs230D1-ferritin at time 0, after incubation at 4°C or room temperature for 24 hours or one cycle of freeze/thaw and (B) SDS-PAGE. (C) Individual amounts of total protein recovered by size exclusion chromatography at time 0, and after incubation at 4°C or room temperature for 24 hours, and one cycle of freeze/thaw, median and 95% confidence interval shown, p-values determined by Friedman test with Dunn's multiple comparisons test.

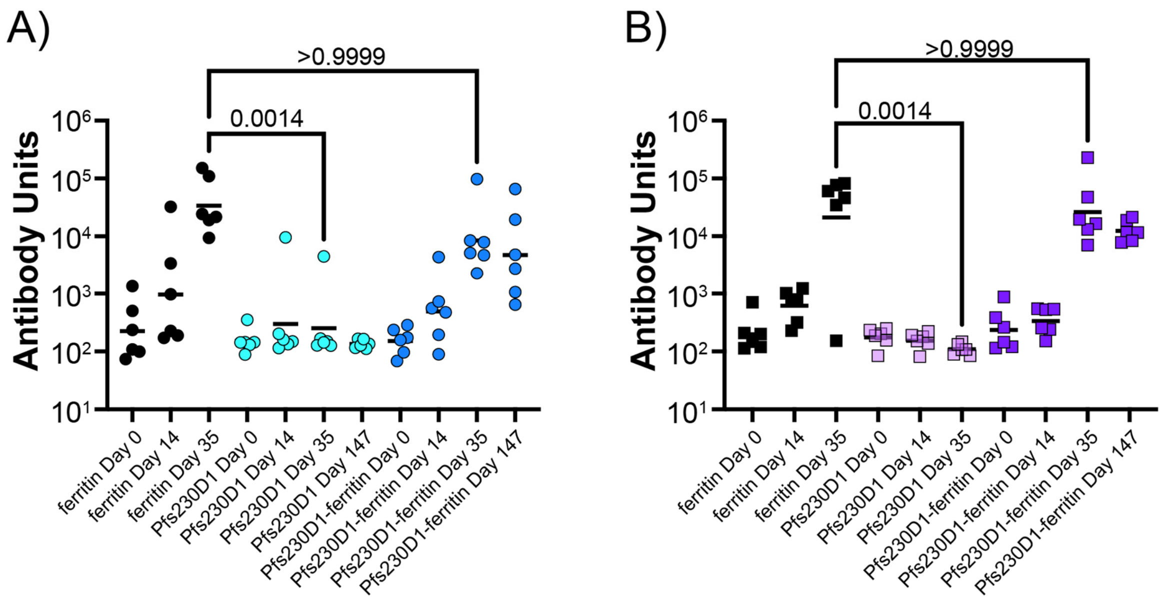

Figure S3. Ferritin specific titers induced by immunization with Pfs230D1, Pfs230D1-ferritin, and ferritin adjuvanted with either (A) Alhydrogel or (B) AddaS03, bars = median, p-values determined by a Kruskal-Wallis test followed by Dunn's multiple comparisons.

Table S1. Pfs230D1 and ferritin median titers over time.

	Median Pfs230D1 Titers			Median ferritin Titers		
	**Day 14**	**Day 35**	**Day 147**	**Day 14**	**Day 35**	**Day 147**
**Pfs230D1/Alhydrogel**	852	67,107	31,600	155	146	131
**Pfs230D1-ferritin/Alhydrogel**	2,789	35,080	14,928	518	6,512	3,772
**ferritin/Alhydrogel**	47	50		600	23,010	
**Pfs230D1/AddaS03**	20	111		166	108	
**Pfs230D1-ferritin/AddaS03**	2,958	228,250	35,287	392	18,045	11,653
**ferritin/AddaS03**	36	40		782	53,300	

Table S2. Transmission reducing activity and the 95% confidence intervals in individual assays for all samples.

	Sample name	Sample, Day Collected	IgG conc [ug/mL]	% Inhibition			
				Estimate	95% CI Low	95% CI High	p-value
SMFA #364	ferritin Alhydrogel	35	3,000	2.1	-122.4	59.8	0.975
	ferritin AddaS03	35	3,000	-49.9	-244.7	37.4	0.344
	Pfs230D1Alhydrogel	35	3,000	98.8	94.8	99.6	0.001
	Pfs230D1Alhydrogel	35	1,000	99.5	98.7	99.9	0.001
	Pfs230D1Alhydrogel	35	333	91.6	80.1	96.4	0.001
	Pfs230D1Alhydrogel	35	111	-8.4	-146.8	52.9	0.837
	Pfs230D1-ferritin Alhydrogel	35	3,000	99.5	97.6	99.7	0.001
	Pfs230D1-ferritin Alhydrogel	35	1,000	98.1	95.2	99.4	0.001
	Pfs230D1-ferritin Alhydrogel	35	333	81.0	55.8	91.7	0.001
	Pfs230D1-ferritin Alhydrogel	35	111	-25.5	-188.0	43.2	0.566
	Pfs230D1 AddaS03	35	3,000	93.4	85.2	97.2	0.001
	Pfs230D1 AddaS03	35	1,000	61.4	12.8	83.3	0.022
	Pfs230D1 AddaS03	35	333	-18.0	-169.8	47.2	0.707
	Pfs230D1 AddaS03	35	111	-12.6	-160.0	51.5	0.780
	Pfs230D1-ferritin AddaS03	35	3,000	100.0	99.4	100.0	0.001
	Pfs230D1-ferritin AddaS03	35	1,000	100.0	99.4	100.0	0.001
	Pfs230D1-ferritin AddaS03	35	333	99.5	98.2	99.9	0.001
	Pfs230D1-ferritin AddaS03	35	111	89.9	77.3	96.1	0.001
SMFA #367-1	Pfs230D1 AddaS03	35	333	9.3	-93.1	59.5	0.806
	Pfs230D1 AddaS03	35	111	-10.2	-144.4	52.4	0.795
	Pfs230D1-ferritin AddaS03	35	333	99.5	98.6	99.9	0.001
	Pfs230D1-ferritin AddaS03	35	111	86.6	64.0	97.3	0.001
SMFA #377-2	Pfs230D1Alhydrogel	35	333	32.7	-42.8	69.5	0.311
	Pfs230D1Alhydrogel	35	111	3.5	-119.4	57.4	0.915
	Pfs230D1-ferritin Alhydrogel	35	333	49.4	-7.5	77.4	0.080
	Pfs230D1-ferritin Alhydrogel	35	111	-28.1	-182.4	43.1	0.552
SMFA #372-2	Pfs230D1Alhydrogel	147	3,000	70.0	33.9	86.2	0.002
	Pfs230D1Alhydrogel	147	1,000	-6.4	-132.3	52.7	0.884
	Pfs230D1-ferritin Alhydrogel	147	3,000	94.8	87.5	98.3	0.001
	Pfs230D1-ferritin Alhydrogel	147	1,000	69.4	29.5	87.7	0.003
	Pfs230D1-ferritin AddaS03	147	3,000	96.4	91.4	98.7	0.001
	Pfs230D1-ferritin AddaS03	147	1,000	65.8	24.0	85.1	0.005
SMFA #377-2	Pfs230D1Alhydrogel	147	3,000	76.0	45.3	90.7	0.001
	Pfs230D1Alhydrogel	147	1,000	-11.9	-128.8	49.3	0.772
	Pfs230D1-ferritin Alhydrogel	147	3,000	91.3	80.6	96.3	0.001
	Pfs230D1-ferritin Alhydrogel	147	1,000	60.2	12.5	82.1	0.016
	Pfs230D1-ferritin AddaS03	147	3,000	98.9	97.4	99.6	0.001
	Pfs230D1-ferritin AddaS03	147	1,000	73.6	41.8	88.5	0.001
